# Rapid Light-Response Curve of Chlorophyll Fluorescence in Terrestrial Plants: Relationship to CO_2_ Exchange among Five Woody and Four Fern Species Adapted to Different Light and Water Regimes

**DOI:** 10.3390/plants10030445

**Published:** 2021-02-26

**Authors:** Meng-Yuan Huang, Shau-Lian Wong, Jen-Hsien Weng

**Affiliations:** 1 Department of Life Sciences, National Chung-Hsing University, Taichung 40227, Taiwan; hmy6@nchu.edu.tw; 2 Division of Botany, Endemic Species Research Institute, Nantou 552, Taiwan; shaulian@tesri.gov.tw

**Keywords:** electron transport rate, fern, photosynthetic rate, rapid light curve, stomatal conductance, tree

## Abstract

The rapid light response of electron transport rate (*ETR*_R_), obtained from chlorophyll fluorescence parameters by short illumination periods (10–30 s) at each light level, can provide a rapid and easy measurement of photosynthetic light response in plants. However, the relationship between *ETR*_R_ and the steady-state light response of CO_2_ exchange rate (*A*_S_) of terrestrial plants has not been studied in detail. In this study, we compared the *ETR*_R_ and *A*_S_ for five woody and four fern species with different light and/or water adaptations. Under well-watered conditions, a constant temperature (25 °C) and with stomatal conductance (*g*_s_) not being a main limiting factor for photosynthesis, *ETR*_R_ and *A*_S_ were closely related, even when merging data for regression analysis for a species grown under different light conditions and measured under different light intensity and air humidity. However, when *Alnus formosana* was treated with low soil water and air humidity, because of the decrease in *A*_S_ mainly due to stomatal closure, the *ETR*_R_–*A*_S_ relation was not so close. In addition, at both 100 and 2000 μmol m^−2^ s^−1^ photosynthetic photon flux density (PPFD), *ETR*_R_ and *A*_S_ were significantly correlated within a plant group (i.e., woody plants and ferns) regardless of the broad difference in *A_S_* due to different species or environmental factors. The results indicate that the relationship between the *ETR*_R_ and *A*_S_ is varied by species. We concluded that 1) ETR_R_ could reflect the variation in *A*_S_ at each irradiance level within a species under well-watered conditions and 2) *ETR*_R_ at 100 μmol m^−2^ s^−1^ PPFD (as the efficiency of light capture) or 2000 μmol m^−2^ s^−1^ PPFD (as a maximum photosynthetic parameter) could be used to compare the photosynthetic capacity within a plant group, such as woody plants and ferns.

## 1. Introduction

Photosynthesis is a major determinant of biomass production and terrestrial carbon budgets [1]. Sunlight is the energy source of plant photosynthesis; however, the response of photosynthesis to light intensity varies by species and environmental conditions. Plants adapted or acclimated to high light often have a high light compensation point, light saturation point, and maximal photosynthetic rate [1,2,3]. Light-response curves (LC) reveal the photosynthetic properties of plants. They can be used to characterize CO_2_ assimilation, photochemistry, photoacclimation, photoinhibition, and photoprotective mechanisms in different light conditions. LC are widely used to describe the physiological plasticity of plants. Thus, the LC of photosynthesis is fundamental for plant ecophysiological research [1,2,3,4].

Traditionally, the LC of photosynthesis has been measured by the rate of steady-state photosynthesis under a range of relevant light intensity. Thus, the measurement is limited by the long measurement time and cumbersome leaf gas exchange techniques, especially in the field [5]. Recently, chlorophyll fluorescence quenching analysis has been found to be a fast, simple, non-invasive, and reliable method to assess changes in photosystem II (PSII) function under different environmental and physiological conditions [6,7,8]. Among chlorophyll fluorescence parameters, electron transport rate (ETR), calculated from the product of PSII efficiency and absorbed light, expresses the relative rate of electron transport through PSII [9,10]. Two ways to obtain light-response data for ETR are steady-state light curve (SLC) and rapid light curve (RLC) methods. 

ETR obtained by SLC methods (*ETR*_S_) is under steady-state conditions at a given strength of illumination. Because CO_2_ fixation (*As*) is a major sink for electrons from PSII, when *A* is inhibited by environmental and/or physiological factors, leaves may downregulate their PSII efficiency, mainly by xanthophyll-dependent non-photochemical quenching to avoid damage caused by excessively absorbed energy [11,12,13,14]. Even if electrons from PSII have several energy sinks (e.g., photorespiration and the water–water cycle) [15,16,17], the allocation of electron flow between *A* and other alternative sinks remains unchanged under many conditions. Examples are C_4_ plants (with photorespiration mostly restricted) and C_3_ plants under conditions with approximate temperature as well as CO_2_ and O_2_ concentrations but varied light intensity [3,18,19,20]. Because both CO_2_ fixation and photorespiration are major sinks for electrons from PSII in C_3_ plants, the ratio of ETR to *As* (or PSII efficiency/photosynthetic rate per absorbed quantum) greatly increases with decreasing CO_2_ partial pressure [21], increasing temperature [3,22], and O_2_ partial pressure [20] because of increased photorespiration.

In contrast to *ETR*_S_, ETR obtained by RLC methods (*ETR*_R_) involves short illumination periods (10–30 s) at each light level, so the RLC can be measured within 1.5–2 min, but leaves do not achieve steady-state conditions during each light step [23,24]. Nevertheless, *ETR*_R_ can provide reliable information about cardinal points of photosynthesis [5,25]. It can use to investigate short-term responses to rapid changes in the light environment [4]. Aquatic photosynthetic organisms often show a parallel change in light responses of *ETR*_R_ and steady-state photosynthetic rate (*A*_S_); thus, *ETR*_R_ is widely used to assess the photosynthetic activity and biomass productivity [26,27,28,29] and to investigate light acclimation [30,31,32,33].

For terrestrial plants, *ETR*_R_ is used to study environmental acclimation [23,34,35,36,37], stress responses [35,38,39,40,41], and estimate photosynthetic efficiency [25,42]. However, in addition to irradiance, stomatal conductance (*g*_s_) is another important limiting factor in the photosynthesis of terrestrial plants. To prevent water loss and facilitate CO_2_ diffusion to mesophyll cells, guard cells may monitor the plant water status and the CO_2_ demand from the mesophyll [1,43]. Stomatal behavior is influenced strongly by water and light conditions. In general, *A* and *g*_s_ may decrease with decreasing light intensity [44,45], as well as soil water content [20,46] and air moisture [2,20]. In addition, the response of stomata to environmental and physiological conditions varies among species. For example, stomata of xerophytic species are more sensitive, and those of hygrophytic species are more insensitive to water deficits than are mesophytic species [47,48]. Moreover, ferns have a lower ability to respond to increases in CO_2_ concentration and decreases to water, for lower *As*/*g*_s_ ratio, than angiosperms [49,50]. In higher plants grown under low light and/or in dry seasons, the maximum values of *A*_S_ and *ETR*_R_ may decrease together [35]. However, the induction of *As* and *g*_s_ requires several minutes (e.g., [51,52]), and the time required for these inductions were varied among species with different light-adaptation capabilities [49]. However, during *ETR*_R_ measurement, leaves are exposed to only 10–30 s of actinic light at each step. Thus, the effect of *g*_s_ on *ETR*_R_ may not be as large as on *A*_S_, and the *ETR*_R_–*A*_S_ relation may vary among species.

Studies elucidating the relation of *ETR*_R_ to *A*_S_ or productivity of terrestrial plants are rare [35,36], as are those investigating the effect of *g*_s_ on the *ETR*_R_–*A*_S_ relation among species across a wide taxonomic range and environmental adaptation and acclimation capability. Due to the difference of light adaptation and acclimation, plants could be broadly divided into sun- and shade-tolerant plants as well as xerophytic and hygrophytic species. Plant species adapted to different light and water regimes show differential photosynthetic characteristics. To obtain a simple, fast, non-invasive, and reliable method to assess photosynthesis under different environmental and physiological conditions [49], we compared the *ETR_R_* and *A_S_* for five woody and four fern species with different light and/or water adaptations. In this study, we examined four fern species, three broad-leaved tree species, and two broad-leaved understory shrubs with different light and/or water adaptation capabilities to investigate these aspects.

## 2. Results

**Figure 1:**
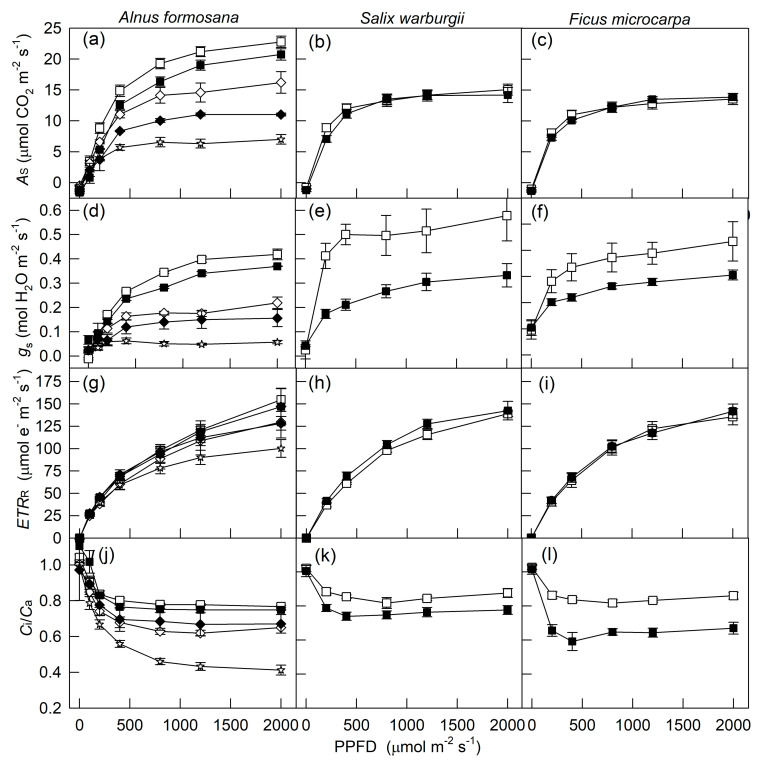
Light-response curves of *A*_s_ (**a**–**c**), *g*_s_ (**d**–**f**), *ETR*_R_ (**g**–**i**), and *C*_i_/*C*_a_ (**j**–**l**) for three tree species measured at 25 °C and 80% (open symbols) and 40% (closed symbols) relative humidity. *A*_S_ and *g*_s_ indicate the net photosynthetic rate and stomatal conductance, respectively, obtained from steady-state light response; *ETR*_R_ indicates electron transport rate obtained from rapid light response; *C*_i_ and *C*_a_ indicate intercellular and atmospheric CO_2_ concentration, respectively, obtained from steady-state light response. Squares, diamonds, and stars *g*_s_ indicate measured under well-watered conditions, mild and severe drought, respectively. Data are mean ± SE.

**Figure 2:**
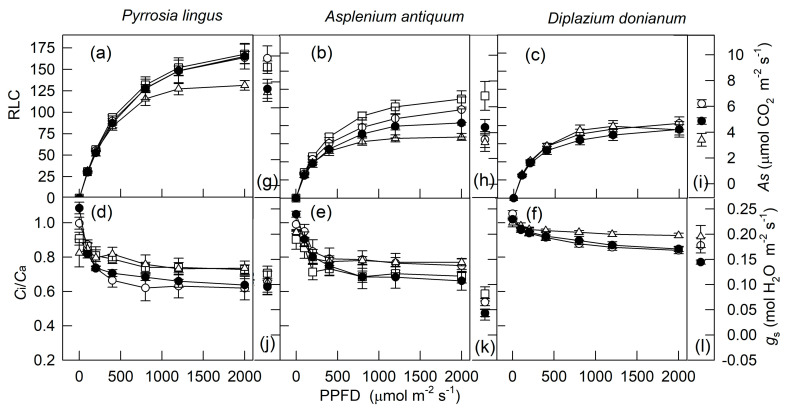
Light-response curves for *ETR*_R_ and *C*_i_/*C*_a_ (**a**–**f**) as well as *A*_S_ and *g*_s_ measured at 2000 μmol m^−2^ s^−1^ photosynthetic photon flux (PPFD; **g**–**l**) for three fern species at 25 °C and 75% (open symbols, data from [3]) and 50% (closed symbols) relative humidity. *A*_S_ and *g*_s_ indicate the net photosynthetic rate and stomatal conductance, respectively, from steady-state light response; *ETR*_R_ indicates electron transport rate from rapid light response; *C*_i_ and *C*_a_ indicate intercellular and atmospheric CO_2_ concentration, respectively. Squares, circles, and triangles indicate cultivated under 100%, 50%, and 10% sunlight, respectively. Data are mean ± SE.

**Figure 3:**
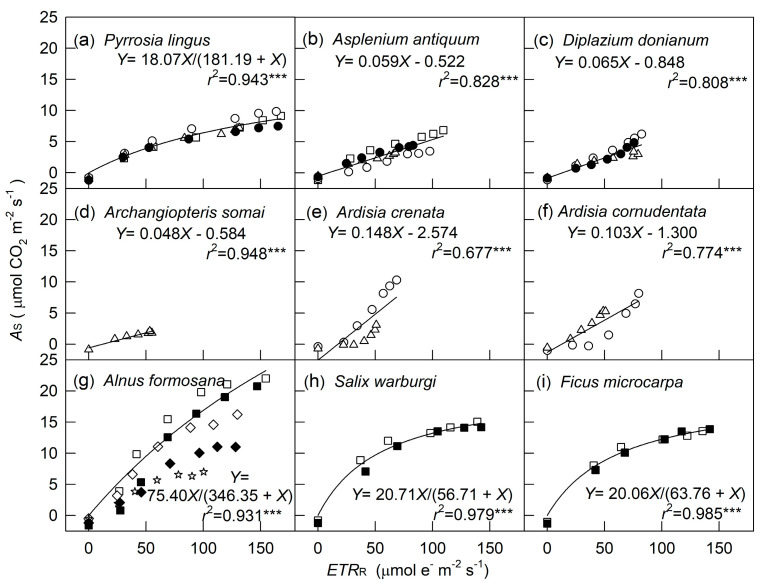
Relationship between net photosynthetic rate from a steady-state light response (*A*_S_) and electron transport rates from a rapid light response (*ETR*_R_) for four ferns (**a**–**d**), two understory shrubs (**e**–**f**), and three tree (**g**–**i**) species. Squares, circles, and triangles indicate cultivated under 100%, 50%, and 10% sunlight, respectively, and measured under well-watered conditions; diamonds and stars (**g**) indicate cultivated under 100% sunlight and measured under mild and severe drought conditions, respectively. Open and closed symbols indicate measured under 75% and 50% relative humidity, respectively, for ferns and understory shrubs, and 80% and 40%, respectively, for trees. *A*_S_ of ferns and understory shrubs measured at 75% relative humidity were from [3]. The regression line in g was fitted for well-watered conditions only. *** is significant at *p* < 0.001.

Figure 1 shows the LCs of *A*_S_, *g*_s_, *ETR*_R_, and intercellular and atmospheric CO_2_ concentration (*C*_i_/*C*_a_) for three tree species measured at 80% and 40% relative humidity (RH). *A*_S_ and *g*_s_ for four ferns and two understory shrubs, measured under well-watered conditions and 75% RH, were described previously [3]. Thus, only the LCs of *ETR*_R_ and *C*_i_/*C*_a_ for *Pyrrosia* *lingus*, *Asplenium* *antiquum,* and *Diplazium* *donianum*, measured at 80% and 40% RH, were selected, as shown in Figure 2a–f. To compare the *A*_S_ and *g*_s_, these two variables for three ferns measured at 2000 μmol m^−2^ s^−1^ photosynthetic photon flux density (PPFD) are also shown in Figure 2g–l. In addition, the relation between *A*_S_ and *ETR*_R_ for all tested species under different PPFD, RH, and soil water conditions is shown in Figure 3. Generally, *A*_S_, *g*_s_, and *ETR*_R_ for all tested species showed a hyperbolic increase with increasing PPFD. However, these LCs varied by species and environmental conditions during cultivation and measurement. Under well-watered conditions, a pioneer tree, *Alnus formosana*, had the highest light saturation point and maximal value of photosynthesis, followed by a hemiepiphytic tree, *Ficus microcarpa*, and a hygrophytic tree, *Salix warburgii*, then by two understory shrubs, *Ardisia crenata* and *Ardisia cornudentata* (Figure 1, Figure 2 and Figure 3 and [3]). In addition, for two understory shrubs, 50% sunlight-grown plants showed a higher maximal value of photosynthesis than 10% sunlight-grown plants (Figure 3e–f). Ferns adapted or acclimated to high light always had a higher light saturation point and maximal photosynthetic rate [3]. Only three trees grown under 100% sunlight and three ferns grown under 50% sunlight were measured under both high and low RH. Under well-watered conditions, the *A*_S_ for *A. formosana* was inhibited only slightly by 40% RH but not for *S. warburgii* and *F. microcarpa*; even the *g*_s_ for these two species was largely inhibited. Both *A*_S_ and *g*_s_ were not affected or were decreased slightly under 50% RH for three ferns. Both *A*_S_ and *g*_s_ were inhibited for *A. formosana* treated with both low soil water content and air moisture. Thus, findings for *A*_S_ and *g*_s_ were similar (Figure 1a,d).

**Figure 4:**
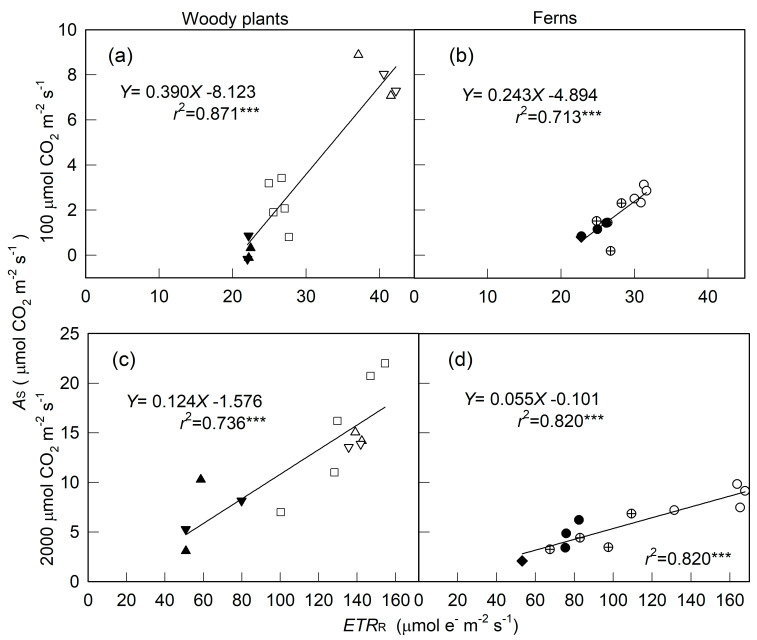
Relationship between net photosynthetic rate from steady-state light response (*A*_S_) and electron transport rates from a rapid light response (*ETR*_R_) for all tested materials at 100 (**a**,**b**) and 2000 (**c**,**d**) μmol m^−2^ s^−1^ PPFD. □ is *Alnus formosana*; △ is *Salix Warburgii*; ▽ is *Ficus microcarpa*; ▲ is *Ardisia crenata*; ▼ is *Ardisia cornudentata*; ○ is *Pyrrosia lingus*; ⊕ is *Asplenium antiquum*; ● is *Diplazium donianum*; ◆ is *Archangiopteris somai*. *A*_S_ for ferns and understory shrubs measured at 75% relative humidity were from [3]. *** is significant at *p* < 0.001.

In contrast to *A*_S_, which for most plants was saturated at 800–1200 μmol m^−2^ s^−1^ PPFD, *ETR*_R_ for high-light- and slight-shade-adapted species did not reach saturation until 2000 μmol m^−2^ s^−1^ PPFD (Figure 1 and Figure 2). Nevertheless, when merging data from the same species measured under different light and moisture conditions, the *A*_S_ for three trees (high-light-adapted) and *P. lingus*, a slight-shade-adapted fern, showed a hyperbolic relation with *ETR*_R_: the *A*_S_–*ETR*_R_ relation could be best fitted by the equation *Y* = a*X*/(b + *X*) (*Y* = *A*_S_, *X* = PPFD, *r*^2^ = 0.943–0.985, *p* < 0.001, Figure 3a,g–i). This relation for the other medium- to heavy-shade-adapted ferns and two understory shrubs was linear (*r*^2^ = 0.677–0.948, *p* < 0.001). The *A*_S_ of *A. formosana* was inhibited largely by low soil water content and low RH, but its *ETR*_R_ was not as inhibited as *A*_S_; thus, the *A*_S_–*ETR*_R_ relation was not as close as for the other tested species. At both 100 and 2000 μmol m^−2^ s^−^*^1^* PPFD, the leaves with high *AS* always had high *ETR_R_*, regardless of species or environmental factors. However, the slope of the *AS–ETR_R_* regression line was higher for woody plants than ferns (Figure 4).

**Figure 5:**
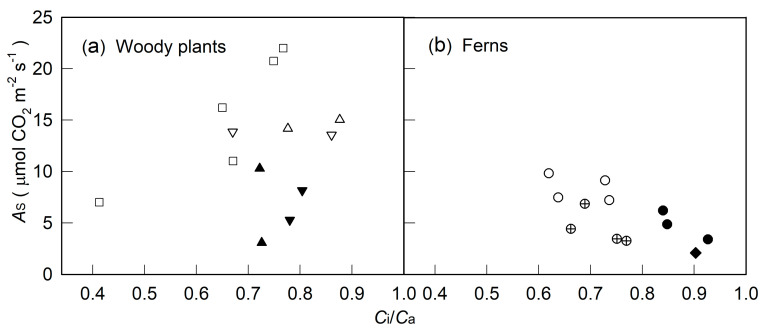
The relationship between net photosynthetic rate (*A*_S_) and the ratio of intercellular CO_2_ concentration (*C*_i_) to atmospheric CO_2_ concentration (*C*_a_) for woody plants (**a**), and ferns (**b**). All data were obtained from the steady-state light response at 2000 μmol m^−2^ s^−1^ PPFD. □ is *Alnus formosana*; △ is *Salix Warburgii*; ▽ is *Ficus microcarpa*; ▲ is *Ardisia crenata*; ▼ is *Ardisia cornudentata*; ○ is *Pyrrosia lingus*; ⊕ is *Asplenium antiquum*; ● is *Diplazium donianum*; ◆ is *Archangiopteris somai*. *A*_S_ for ferns and understory shrubs measured at 75% relative humidity were from [3].

The *C*_i_/*C*_a_ for all measurements decreased with increasing PPFD and stabilized somewhat at about 800 μmol m^−2^ s^−1^ PPFD with most treatments (Figure 1j–l and Figure 2d–f). At 2000 μmol m^−2^ s^−1^ PPFD, the *A*_S_–*C*_i_/*C*_a_ relation could be divided into four groups: (1) four ferns, (2) two understory shrubs, (3) *Ficus microcarpa* and *Salix Warburgii,* and (4) *Alnus formosana* (Figure 5). The *A*_S_ for ferns was decreased, and that for *A. formosana* was increased with increasing *C*_i_/*C*_a_. However, the *A*_S_ for *F. microcarpa* and *S. warburgii* was not affected by *C*_i_/*C*_a_. As well, although the *A*_S_ for two understory shrubs was inhibited by 10% sunlight during growth, their *C*_i_/*C*_a_ was not greatly affected.

## 3. Discussion

The *ETR*_R_–*A*_S_ relation of terrestrial plants has not been studied in detail, especially among species across a wide taxonomic range and environmental adaptation capability. In this study, we compared the *A*_S_ and *ETR*_R_ for five woody and four fern species with different light and/or water adaptations. The obtained data showed a broad range of *A*_S_ because of the specific differences of species and the environmental conditions under which materials were cultivated and measured. Under the steady-state, plants adapted or acclimated to high light always had high values of both light saturation point and maximal photosynthetic rate (Figure 1 and Figure 2 and [3]), which agreed with previous results (e.g., [1,2]).

ETR is calculated as the product of PSII efficiency and absorbed light. Many studies used the empirical mean of α (0.84) to calculate ETR and compare differences in ETR among species [5] and under different growth irradiances [35]. However, the α value may vary by leaf pigment content and anatomical structures. Previously, we examined leaves with a broad range of chlorophyll content (0.18–0.55 g m^−2^) and found a similar association of *A*_S_ and ETR regardless of the use of α = 0.84 or 0.80–0.89 (from an empirical regression equation between α and chlorophyll content) to calculate ETR (Weng et al. unpublished data). In addition, our plants featured no specific anatomical structures. So we chose the empirical mean α of 0.84 [5].

Measurement of SLC requires light steps long enough to allow for stabilization of the photosynthetic processes under each irradiance level. RLC only requires 10 to 30 s at each light level; nevertheless, the difference in *A*_S_ between high- and low-light-grown materials can be defined by *ETR*_R_ [24,31,35]. However, in addition to a long-term photoacclimation status, *ETR*_R_ also depends on the short-term (min) light history of photosynthetic organisms immediately before measurement as well as illumination time for each light level during measurement. Maximum *ETR*_R_ value is very low after long-term dark adaptation, but when organisms are exposed to light immediately before RLC measurement, maximum *ETR*_R_ increases with increasing illumination time and to a stable level within 8 to 15 min of illumination [23]. In addition, maximum *ETR*_R_ increases with increasing light intensity immediately before measurement [30] but may decrease under high light (2000 μmol m^−2^ s^−1^ PPFD) pre-irradiance [23]. During measurement, maximum *ETR*_R_ increases with increasing illumination time at each light level [24,53].

Our materials were acclimated to 1 to 3 levels of light intensity for at least five months. To minimize the effects of light history immediately before measurement on the *ETR*_R_ and *A*_S_, we used overnight dark-adapted leaves for measurement of *ETR*_R_ and then measured leaves were kept in the dark until measurement of *A*_S_. Even for leaves not exposed to light until the measurement and with short (10-s) steps of increasing irradiance, *ETR*_R_ was still high (50–168 μmol e^-^ m^−2^ s^−1^ for all materials at 2000 μmol m^−2^ s^−1^ PPFD) and closely related to *A*_S_ (Figure 3 and Figure 4). Thus, *ETR*_R_ could reflect the broad range in *A*_S_ among different species and environmental conditions.

Photosynthesis is limited by both stomatal and non-stomatal factors. The former is associated with decreased leaf *C*i caused by stomata closure, and the latter with a decrease in photochemical efficiency and CO_2_ fixation [1,46]. The *g*_s_ value often decreases with decreasing light intensity [44,45] as well as soil water content [20,46,54] and air humidity [2,20]. However, photosynthetic electron transport and CO_2_ fixation are also inhibited by low light [3,5,23] and low soil water content [20,54]. In the present study, all *A*_S_ and *g*_s_ values decreased with decreasing light intensity (Figure 1 and Figure 2). However, the effect of low RH on *g*_s_ varied by species. Under well-watered conditions, the *g*_s_ for *F. microcarpa* and *S. warburgii* was largely inhibited by 40% RH (Figure 1e,f). Nevertheless, *A*_S_ did not differ greatly at 80% and 40% RH for two trees (Figure 1b,c). In contrast, only *A. formosana* was treated with low soil water content, and its *A*_S_, *g*_s_, and *ETR*_R_ values were markedly inhibited (Figure 1a,d,g). Thus, we revealed a combination of stomatal and nonstomatal effects on photosynthesis.

At 2000 μmol m^−2^ s^−1^, the *A*_S_ for four ferns decreased with increasing *C*_i_/*C*_a_ when *A*_S_ was affected by specific differences of species and environmental conditions during cultivation and measurement (Figure 5b). Based on the relation between *C*_i_/*C*_a_ and *A*_S_ [46,51,55], indicating non-stomatal factors were the main cause of the difference in *A*_S_ for the four ferns. In contrast, the *A*_S_ for *A. formosana* increased with increasing *C*_i_/*C*_a_ under low RH and low soil water content (Figure 5a). The decrease in *A*_S_ was explained mainly by stomatal closure (Figure 1d). The *A*_S_ for *F. microcarpa* and *S. warburgii* was not affected by *C*_i_/*C*_a_ (Figure 5a), so neither stomatal nor non-stomatal factors were a main limiting factor for the *A*_S_ for these two species, even if their *g*_s_ value was markedly limited by low RH (Figure 1e,f). As well, the *C*_i_/*C*_a_ for two 10%-sunlight-grown understory shrubs was not affected by decreasing *A*_S_ (Figure 5a), so *A*_S_ decreased with decreasing *g*_s_.

*ETR*_R_ is related to environmental acclimation [23,34,35,36] and stress responses [35,38,39] of terrestrial plants but is rarely used to elucidate the relation with the photosynthetic rate [35]. When data in Figure 1, Figure 2 and Figure 3 and Figure 5 were combined, stomatal limitation played an important role in affecting the *ETR*_R_–*A*_S_ relation within a species. When stomatal closure was not a main limiting factor for photosynthesis, *ETR*_R_ and *A*_S_ were closely related across a wide range of PPFD, even when merging data for a species grown under different light conditions and measured under different light intensity and RH (Figure 3). This finding may explain why almost all research involving *ETR*_R_ to assess photosynthetic activity or biomass productivity is limited to species without stomatal limitations to CO_2_ uptake, such as algae [26,27,29] and coral [28]. However, the induction of *A* and ETR requires several minutes to reach stability (e.g., [51,52]), whereas leaves are exposed to only 10–30 s of actinic light at each step during *ETR*_R_ measurement. Thus, *ETR*_R_ represents only the potential response of steady-state photosynthesis under a range of light conditions [30].

Our results indicate that the *ETR*_R_–*A*_S_ relationship varies by species. The increase in LC in the light-limiting region, as well as light-saturation and maximum photosynthetic variables, have been used for research into plant ecophysiology [24,30,31,35]. Because the *ETR*_R_ for some species did not reach saturation until 2000 μmol m^−2^ s^−1^ PPFD, we could not compare the light-saturation variable among species. Here, we used only data obtained at 100 μmol m^−2^ s^−1^ PPFD for the efficiency of light capture and data obtained at 2000 μmol m^−2^ s^−1^ PPFD as a maximum photosynthetic variable to elucidate the interspecific relationship between *ETR*_R_ and *A*_S_. A significant *ETR*_R_–*A*_S_ relation could be found within a plant group (i.e., woody plants and ferns) (Figure 4). Therefore, the *ETR*_R_ value obtained at both 100 and 2000 μmol m^−2^ s^−1^ PPFD could be used to compare the photosynthetic capacity within the same plant group, regardless of a broad difference in *A*_S_ due to different species or environmental factors, with the empirical mean of α (0.84) used to calculate ETR for all tested materials. Moreover, slopes were higher for woody plants than ferns for both *AS*–*ETR_R_* (Figure 4) and *AS*–*ETR*_S_ [3] on regression analysis, which might somewhat be caused by the difference of light absorptivity of leaf among species. However, even with α value changed from 0.80 to 0.89 (chlorophyll content from 0.18 to 0.55 g m^−2^, Weng et al. unpublished data), there was only a 1.1-fold difference between ETR with α = 0.81 and 0.89 used for calculation. However, the difference in slopes for the *AS*–*ETR_R_* regression between woody plants and fern was much higher than 1.1-fold (1.6- and 2.3-fold at 100 and 2000 μmol m^−2^ s^−1^ PPFD, respectively, Figure 4). Thus, we prefer to explain that tested woody species could share more electrons for CO_2_ fixation at a given ETR level than ferns. This finding might be due to differences in allocation portion between CO_2_ fixation and alternative electronic pathways [19,22], such as photorespiration [15], water–water cycle [16], and cyclic electron flow within PSII [17] as well as nitrogen [56] and sulfur [57] assimilation.

In the present study, we found that 1) *ETR*_R_ could reflect the variation in *A*_S_ at each irradiance level within a species under well-watered conditions and 2) *ETR*_R_ obtained at 100 μmol m^−2^ s^−1^ PPFD (as the efficiency of light capture) or 2000 μmol m^−2^ s^−1^ PPFD (as a maximum photosynthetic parameter) could be used to compare the photosynthetic capacity within a plant group, such as woody plants and ferns. Because *ETR*_R_ can be measured within 1.5–2 min, it might be a useful tool for ecophysiological research. However, we investigated only five woody plants and four fern species. The number of species may not be enough to argue the taxonomic distinctions, and more comparisons might be needed. In addition, photorespiration is another major sink for electrons from PSII in C_3_ plants. The A_S_/*ETR*_R_ ratio may vary on changing the CO_2_ and O_2_ concentration as well as temperature because the allocation of electrons between CO_2_ fixation and photorespiration may vary.

## 4. Materials and Methods

### 4.1. Plant Materials

We examined 4 fern species with different light adaptation (ranked from high to low light adaptation: *Pyrrosia lingus* (Thunb.) Farw., *Asplenium antiquum* Makino, *Diplazium donianum* (Mett.) Tard. -Blot., and *Archangiopteris somai* Hayata), 3 broad-leaved tree species with different water adaptation (*Alnus formosana* (Burkill) Makino, a pioneer tree; *Salix warburgii* O. Seem., a hygrophyte, and *Ficus microcarpa* L., a hemiepiphyte) and 2 broad-leaved understory shrubs (*Ardisia crenata* Sims. and *Ardisia cornudentata* Mez.) in this study [48,49,58]. In addition, *A. formosana* and *S. warburgii* are usually distributed near the rivers or in gullies; *P. lingus* and *F. microcarpa* can survive in the dry environment; whereas *D. donianum*, *Arc*. *Somai,* and *S. warburgii* are sensitive to drought [3]. Four ferns (adult plants, about 30 cm tall), 2 understory shrubs (adult plants, about 60 cm tall), and *A. formosana* (1- to 2-year-old seeding, about 30–50 cm tall) were the same as we used previously, and collected from central Taiwan [3]. The other 2 trees were only used in the present study and were propagated from cuttings (about 30–50 cm tall). All plants were collected in March and then transplanted to pots (16-cm diameter, 12-cm depth, 1 plant per pot for the five woody species and *As. antiquum*, and 1 rhizome with 3–4 leaves per pot for the other 3 ferns) filled with organic soil and maintained outdoors in the nursery of the Endemic Species Research Institute, Chichi Township, Nantou County, Taiwan (23°49′ N, 120°48′ E, 250 m a.s.l.). Materials were regularly watered and fertilized (half-strength Hoagland’s nutrient solution per month) and received up to 3 levels of light intensity (i.e., 100%, 50%, and 10% (beneath shade cloth)), according to the light condition of their habitat, i.e., 3 trees received 100% sunlight; 2 slight- to medium-shade ferns, *P. lingus* and *As. antiquum*, received 100%, 50%, and 10% sunlight; 1 medium-to-heavy shade fern, *D. donianum*, and 2 understory shrubs received 10% and 50% sunlight; and 1 heavy-shade fern, *Arc. somai* received 10% sunlight. The average elevation and temperature were about 250 m and 20 °C. The average annual rainfall and air humidity were about 2200 mm and 80%. During the growth period of the materials (March–November), the average hourly values of daily maximum photosynthetic photon flux density (PPFD) ranged from 1296−1456 μmol m^−2^ s^−1^ (Mar.−Aug.) and 1150−770 μmol m^−2^ s^−1^ (Sept.−Nov.) (data from the Endemic Species Research Institute). Only *A. formosana* was treated with mild and severe drought immediately before photosynthetic measurement by withholding water, until *A*_S_ values were reduced to about 70% and 30%, respectively, of the maximum (*A*_S_ under well-watered conditions: 100%) [54].

### 4.2. Measurements

Measurements were carried out from September to November in a laboratory at the Endemic Species Research Institute. At nightfall of 1 day before the measurement, potted materials were dark-adapted overnight (room temperature about 25 °C). On the next day, fully expanded younger leaves were selected for measurements. First, the measurement of *ETR*_R_ was at dawn at room temperature and involved the software of the PAM-2000 fluorometer (Walz, Effeltrich, Germany). Nine steps of active light from about 60–2300 μmol m^−2^ s^−1^ PPFD were applied at each irradiation step for 10 s [23,34,35]. The actual (*F*) and maximal (*F*_m_’) levels of fluorescence were measured at the end of each irradiance level. The *F* was determined under each PPFD level, and the *F*_m_’ was determined by applying a 0.8-s pulse of saturating flashes of approximately 6000 μmol quanta m^−2^ s^−1^. Actual PSII efficiency (Φ_PSII_) was calculated as (*F*_m_’−*F*)/*F*_m_’, and *ETR*_R_ was calculated as Φ_PSII_ × PPFDD × 0.5 × α [8]. We used the mean value of leaf absorption (α) of 0.84 for green leaves [59] (see Discussion section). *ETR*_R_ at 200, 400, 800, 1200, and 2000 μmol m^−2^ s^−1^ PPFD was calculated from linear interpolation between the 2 nearest values. After the measurement of *ETR*_R_, the measured leaves were kept in the dark until the measurement of the steady-state light response of CO_2_ exchange. From 09:30 h to 15:00 h, photosynthesis and stomatal conductance were measured by use of a portable, open-flow gas exchange system (LI-6400, LI-COR, Lincoln, NE, USA), and an integrated fluorescence LI-6400-40 chamber head stepwise from low to high levels of PPFD (i.e., 0, 100, 200, 400, 800, 1200, and 2000 μmol m^−2^ s^−1^). The values of *A*_S_ (net photosynthetic rate), *g*_s_, and intercellular CO_2_ concentration/ambient CO_2_ concentration (*C*_i_/*C*_a_) were recorded when the gas exchange was stable (about 4 min in the dark and 10–20 min under each level of illumination). Throughout the measurements, leaf temperature and CO_2_ concentration were kept at 25 °C and 350–400 μmol mol^−1^ (no control), respectively, for all materials. Relative humidity (RH) in the chamber was taken at 75% and 50% (air entering chamber controlled by passing temperature-controlled water) for ferns and understory shrubs, and 80% and 40% for trees.

### 4.3. Statistical Analysis

Four to 6 fully expanded younger leaves from 4 plants of each species grown in each light condition were measured. Each leaf was taken as 1 replicate for statistical analyses. The results are expressed as the mean ± standard error (SE). The light-response curve of photosynthetic rate was fitted by sigmoidal or hyperbolic equations. Data were analyzed by linear or curve–linear regression. All statistical analyses involved the use of Sigma Plot v10.0.
